# Association between anti-Mullerian hormone and antithyroid antibodies detectability and positivity in women assisted at a fertility clinic

**DOI:** 10.5935/1518-0557.20250032

**Published:** 2025

**Authors:** Yuri Ian Lima de Oliveira, Arnaldo Couto, Paulo Gallo de Sá, Lenora Maria Camarate Silveira Martins Leão, Ana Beatriz Winter Tavares

**Affiliations:** 1 Postgraduate Program in Clinical and Experimental Physiopathology (Fisclinex), Faculty of Medical Sciences, Rio de Janeiro State University, Rio de Janeiro, Brazil; 2 FERTGROUP - Vida Assisted Reproduction Center, Rio de Janeiro, Brazil; 3 Pharmacy Department - Rio de Janeiro State University, Rio de Janeiro, Brazil; 4 Department of Gynecology - Faculty of Medical Sciences - Rio de Janeiro State University, Rio de Janeiro, Brazil; 5 Endocrine Service - Department of Internal Medicine - Faculty of Medical Sciences - Rio de Janeiro State University, Rio de Janeiro, Brazil

**Keywords:** Anti-Mullerian hormone, thyroid autoimmunity, antithyroid antibodies, assisted reproductive technique, antithyroglobulin, infertility, female infertility

## Abstract

**Objective:**

To evaluate the association between antithyroid antibodies detectability and positivity and ovarian reserve. Additionally, we aimed to evaluate the correlation between TSH levels and parameters of ovarian reserve [anti-Mullerian (AMH) levels and antral follicle count (AFC)].

**Methods:**

Subjects were women seeking assisted reproductive therapy and oocyte cryopreservation. AMH, AFC and antithyroid antibodies were determined. In a subgroup of patients, antithyroid antibodies were stratified into categories for the association analyses. The reference values were as follows: antithyroid peroxidase antibody (TPOAb): undetectable (<9 IU/mL), low detectable (9-21 IU/mL), high detectable (22-34 IU/mL), and positive (> 34 IU/mL); antithyroglobulin antibody (TgAb): undetectable (< 10 IU/mL), low detectable (10-60 IU/mL), high detectable (61-115 IU/ mL), and positive (>115 IU/mL). AMH levels < 1.1 ng/mL and/or AFC < 7 follicles were considered to indicate low ovarian reserve.

**Results:**

A total of 453 patients were enrolled. 24.4% had at least one positive antithyroid antibody. Low detectable values for TPOAb and TgAb were found in 42.3% and 66.9%, respectively. TPOAb and TgAb levels were not statistically different between women with AMH < or ≥ 1.1 ng/mL; there was also no statistically significant difference between antithyroid antibodies levels and AFC < or ≥ 7 follicles. AMH levels did not show significant association with detectability nor positivity of the antithyroid antibodies. No statistically significant correlation was observed between AMH and TSH nor AFC and TSH.

**Conclusions:**

Although the detectability of TPOAb has been associated with different outcomes, TPOAb and/or TgAb were not associated with ovarian reserve in our study.

## INTRODUCTION

Ovarian function and reserve can be compromised by autoimmune diseases, among which Hashimoto’s thyroiditis is the most common in women during the reproductive age, with a prevalence of up to 20% (Vanderpump *et al.*, 1995; Aoki *et al.*, 2007), while hypothyroidism is involved in the pathogenesis of 27% of premature ovarian failure (POF) cases (Anasti, 1998).

Chronic reduction in thyroid hormone levels results in a wide spectrum of reproductive changes in addition to POF, such as abnormal folliculogenesis, changes in the ovulatory cycle, and a lower fertilization rate (Aghajanova *et al.*, 2009; Vissenberg *et al.*, 2015). Thyroid autoimmunity, independent of thyroid dysfunction, has already been associated with lower antral follicle counts (Korevaar *et al.*, 2018).

The assessment of ovarian reserve is well established based on the dosage of anti-Mullerian hormone (AMH), which is secreted by the granulosa cells of the ovarian follicles and has a stable value regardless of the stage of the cycle or the use of hormonal contraceptive methods (Nardo *et al.*, 2009; di Clemente *et al.*, 2021). The main factor directly related to lower AMH secretion is advancing age, with a reduction of approximately 5-7 pmol/L every 3-5 years during the fertile period (Ortega *et al.*, 1990). Several studies have evaluated the association between AMH and thyroid function and/or autoimmunity, but the results have been discordant (Kuroda *et al.*, 2015; Polyzos *et al.*, 2015; Osuka *et al.*, 2018; Morales-Martínez *et al.*, 2021; Öztürk Ünsal *et al.*, 2021). Lower ovarian reserve has already been demonstrated in women over 35 years of age with subclinical hypothyroidism (Rao *et al.*, 2020). In a cohort of euthyroid women with reduced ovarian reserve or unexplained infertility, antithyroid peroxidase antibody (TPOAb) positivity was associated with a lower antral follicle count (AFC) (Korevaar *et al.*, 2018).

Detectable TPOAb levels, even below positive values, have already been associated with increased mortality and the clinical applicability of detecting TPOAb levels has been discussed as a potential marker of low-grade inflammation (Khan *et al.*, 2022). The detectability of antithyroid antibodies in women and its association with ovarian reserve has not been studied yet.

The main objective of the current study was to evaluate the association between ovarian reserve and antithyroid antibodies detectability and positivity in a large sample of women seeking assisted reproduction therapy (ART) or oocyte cryopreservation. Additionally, we aimed to evaluate the correlation between TSH levels and parameters of ovarian reserve (AMH and AFC).

## MATERIAL AND METHODS

### Study design and subjects

Observational study based on medical records, carried out in women who looked for Vida Assisted Reproductive clinic - Fertility Center (Rio de Janeiro, Brazil) and went through the process of ovarian stimulation and oocyte retrieval from July 2021 to July 2023. Inclusion criteria were: women attended in the outpatient clinic with baseline exams before ovarian stimulation. Exclusion criteria were: women with thyroid dysfunction, patients using medications that may influence the hypothalamic-pituitary-thyroid axis, such as glucocorticoids in supraphysiological doses; or that could interfere with thyroid hormone metabolism (lithium, amiodarone and aspirin in anti-inflammatory dosages).

Clinical data including chronological age, main reasons for medical appointment, comorbidities and current use of medications were obtained from records of the first care. Body mass index (BMI) was calculated by the formula: weight/height^2^ (Kg/m^2^). All the serum tests were performed in a single laboratory. Hormonal data were measured by eletrochemiluminescence immunoassay (Elecsys, Mannheim-Germany) in blood samples collected between the third and fifth day of a spontaneous or induced menstrual cycle, approximately 30 days before the start of ovarian stimulation. The reference range were: FSH 3.5 - 12.5 mIU/L; TSH 0.4 - 4.3 mIU/L; and free thyroxine (FT4) 0.7 - 1.9 ng/dL.

AMH levels <1.1 ng/mL and/or AFC <7 follicles were considered low ovarian reserve, according to the consensus of the Brazilian Society of Human Reproduction (Miklos *et al.*, 2019). TPOAb and antithyroglobulin antibody (TgAb) levels were also assessed using an electrochemiluminescenceimmunoassay (Elecsys - Mannheim-DE). The TPOAb lower and upper detection limit were, respectively, 9 IU/mL and 600 IU/mL; the TgAb lower and upper detection limits were 10 and 4000 IU/mL.

Antithyroid antibodies were stratified into categories according to their values: a) TPOAb: undetectable (<9 IU/mL), low detectable (9-21 IU/mL), high detectable (22-34 IU/mL), and positive (>34 IU/mL); and b) TgAb: undetectable (<10 IU/mL), low detectable (10-60 IU/mL), high detectable (61-115 IU/mL), and positive (>115 IU/mL).

Ovarian stimulation was started after a baseline transvaginal ultrasound (also performed in the early follicular phase) to determine AFC and rule out potential contraindications to ART, such as ovarian cysts or spontaneous pregnancy. 2D transvaginal ultrasound was performed with a 9MHz endocavitary probe. In Vida Assisted Reproduction clinic the following protocols were used: FISCHER protocol (Fischer *et al.*, 2019) using either dydrogesterone as a pituitary blockade or GnRH antagonist; and a flexible antagonist - to start GnRH antagonist when the largest ovarian follicle reached 14 mm in average diameter (Grzechocińska *et al.*, 2003). AFC and oocyte retrieval at an appropriate time were performed by a specialist medical doctor in assisted human reproduction.

The study was approved by the Institutional Review Board of Pedro Ernesto University Hospital (Rio de Janeiro State University) under the number 031550/2021, and the study was conducted in accordance with the Declaration of Helsinki. Written informed consent was obtained from all patients prior to study enrollment. This trial was registered at Clinical Trials Registry as NCT06394466.

### Statistical analysis

The study variables were categorized and analyzed to identify statistical differences between the groups. Comparisons of differences between the proportions (relative frequencies) of categorical variables were made using the Chi-square test (ᵪ2) or Fisher’s exact test (if applicable). To assess the magnitude of association, unconditional logistic regression was performed, obtaining odds ratios (OR) with their respective 95% confidence intervals. Furthermore, to evaluate possible correlations between the quantitative variables, scatter plots were constructed, with the degree of correlation using the Pearson coefficient (when there was normality in the data distribution) and the Spearman coefficient (when there was no normality in the data distribution). Normality in the distribution of data in quantitative variables was observed using the Kolmogorov-Smirnov and Shapiro-Wilk tests.

All data analyzes were performed using the statistical software SPSS 26.0 (Statistical Package for Science - Chicago, IL, 2019) and differences with *p*<0.05 were considered statistically significant.

## RESULTS

This study included 453 women. Clinical, laboratory and ultrasound characteristics of the studied population are presented in Table 1. Women presented a median age of 38 years old (IQR 35-44) and had a BMI that was predominantly in the normal/overweight range. According to medical appointments, endometriosis was the main cause for seeking ART (almost 70%) while 30.5% of women had more than one cause.

**Table 1:** Clinical, laboratory and ultrasound characteristics of the studied population.

Characteristics (n=453)	
Age (years) [median (IQR)]	38 (IQR 35-44)
BMI (kg/m^2^), n (%) Underweight (≤18.5) Healthy weight (18.6-24.9) Overweight (25-29.9) Obesity (≥30) Not informed	7 (1.6) 244 (53.8) 140 (30.9) 36 (7.9) 26 (5.8)
Causes of appointments, n (%) Endometriosis Low ovarian reserve Male factor Tubal factor Social cryopreservation of oocytes Other causes	314 (69.3) 99 (22) 99 (22) 48 (10.7) 41 (9) 70 (15.5)
TSH (mIU/L)	1.67 (IQR 1.22-2.4)
FT4 (ng/dL)	1.27 (IQR 1.16-1.4)
FSH (mIU/L)	8.0 (IQR 5.7-12.6)
AMH (ng/mL)	1.47 (IQR 0.71-3.05)
AFC (number of follicles)	11 (IQR 7-17)

BMI: Body mass index; IQR: Interquartile range; TSH: Thyroid stimulating hormone; FT4: Free thyroxine; FSH: Follicle-stimulating hormone; AMH: Anti-Mullerian hormone; AFC: Antral follicle count

As expected, AMH and AFC were significantly lower in women > 35 years than in those ≤35 years [median AMH 1.21 ng/mL (IQR 0.77-2.0) *versus* 2.9 ng/mL (IQR 1.165-4.8), respectively, and median AFC 10 follicles (IQR 6-14) *versus* 17 follicles (IQR 10-28), respectively] (*p*=0.001 for both).

Based on the total sample, 106 women (24.4%) tested positive for at least one antithyroid antibody. Isolated positive TPOAb was found in 14%, while 11.6% had isolated positive TgAb. In 185 women, antithyroid antibodies were registered only as positive (4%) or negative (96%) - thus, they were not included in the thyroid autoimmunity stratification for association analysis. In the last 260 women, who presented numerical values for antithyroid antibodies, most patients had low detectable values for TPOAb and TgAb (42.3% and 66.9%, respectively).

TPOAb and TgAb levels were not statistically different between women with normal AMH levels and those with AMH levels compatible with low ovarian reserve. There was also no statistically significant difference between the antithyroid antibodies levels and normal or reduced AFC (Table 2).

**Table 2:** Anti-peroxidase and antithyroglobulin antibodies levels according to Anti-Mullerian Hormone and Antral Follicle Count.

Variables	Anti-Mullerian Hormone (AMH)		p	Antral Follicle Count (AFC)		p
	Normal (≥1.1 ng/mL)	Reduced (<1.1 ng/mL)		Normal (≥7)	Reduced (<7)	
**TPOAb**	**n=149**	**n=98**	0.41	**n=59**	**n=201**	0.35
Undetectable (<9 IU/mL) Low detectable (9-21 IU/mL) High detectable (22-34 IU/mL) Positive (>34 IU/mL)	62 (41.6%) 60 (40.3%) 6 (4%) 21 (14.1%)	31 (31.6%) 47 (48%) 6 (6.1%) 14 (14.3%)		18 (30.5%) 28 (47.5%) 2 (3.4%) 11 (18.6%)	81 (40.3%) 82 (40.8%) 12 (6%) 26 (12.9%)	
**TgAb**	**n=149**	**n=99**	0.70	**n=59**	**n=201**	0.33
Undetectable (<10 IU/mL) Low detectable (10-60 IU/mL) High detectable (61-115 IU/mL) Positive (>115 IU/mL)	28 (18.8%) 102 (68.5%) 4 (2.7%) 15 (10.1%)	17 (17.2%) 64 (64.6%) 4 (4%) 14 (14.1%)		13 (22%) 34 (57.6%) 21 (3.4%) 10 (16.9%)	34 (16.9%) 140 (69.6%) 6 (2.9%) 21 (10.4%)	

TPOAb: antithyroid peroxidase antibody; TgAb: antithyroglobulin antibody.

There was no association between AMH levels and detectable categories of TPOAb or TgAb or between AMH levels and positivity of the antithyroid antibodies, even after adjustment for age and endometriosis (Table 3).

**Table 3:** Association between Anti-Mullerian Hormone levels and detectable/positive antithyroid antibodies.

Variables	Anti-Mullerian Hormone (AMH)		p	OR (95% CI)	Adjusted OR^*^ (95% CI)
	Normal (≥1.1 ng/mL)	Altered (<1.1 ng/mL)			
**TPOAb**	**n=87**	**n=67**	0.82		
Positive (>34 IU/mL) High detectable (22-34 IU/mL) Low detectable (9-21 IU/mL)	21 (24.1%) 6 (6.9%) 60 (69%)	14 (20.9%) 6 (9%) 47 (70.1%)		1.00 1.50 (0.40 - 5.60) 1.18 (0.54 - 2.55)	1.00 2.00 (0.48 - 8.21) 1.30 (0.58 - 2.92)
**TgAb**	**n=121**	**n=82**	0.52		
Positive (>115 IU/mL) High detectable (61-115 IU/mL) Low detectable (10-60 IU/mL)	15 (12.4%) 102 (84.3%) 4 (3.3%)	14 (17.1%) 34 (78.0%) 4 (4.9%)		1.00 1.07 (0.22 - 5.12) 0.67 (0.30 - 1.48)	1.00 1.08 (0.20 - 5.75) 0.51 (0.22 - 1.20)

* Adjusted OR for TPOAb, TgAb, age and endometriosis.

TPOAb: antithyroid peroxidase antibody; TgAb: antithyroglobulin antibody.

**Figure 1:**
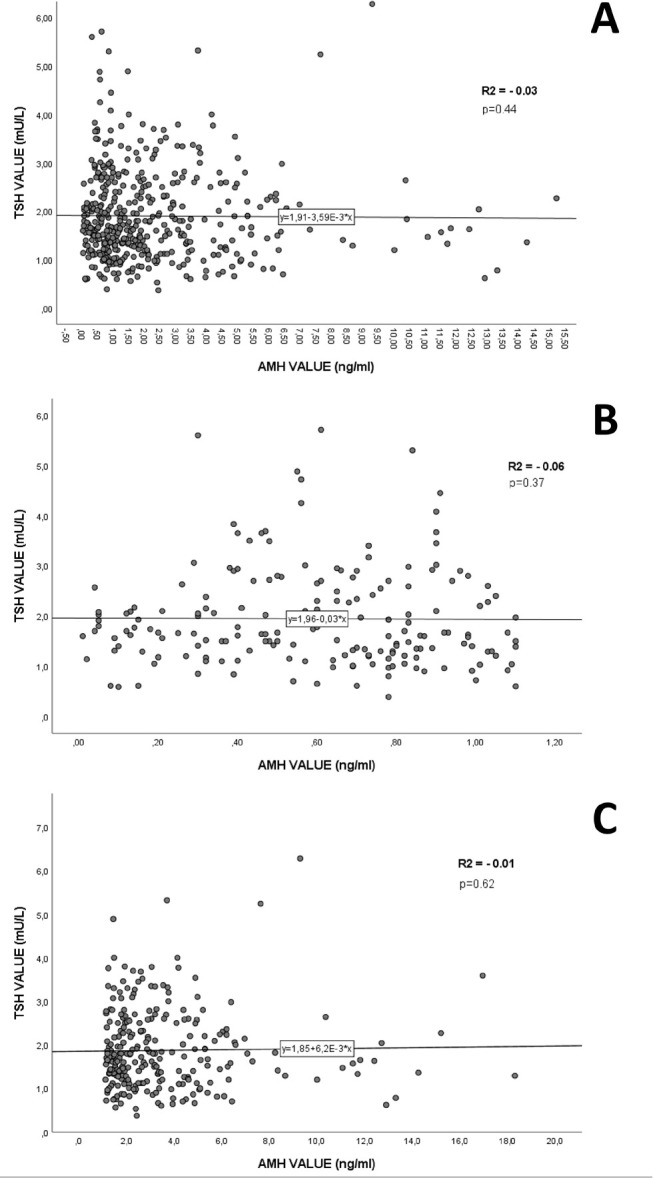
Assessment of correlation between Anti-Mullerian Hormone (AMH) and TSH levels. (a) General correlation. (b) Correlation stratified by AMH levels < 1.1 ng/mL. (c) Correlation stratified by AMH levels ≥ 1.1 ng/mL.

AMH levels and BMI were not statistically correlated (*p*=0.47). No statistically significant correlation was observed between AMH and TSH (*p*=0.44), even when AMH levels were categorized as <1.1 ng/mL (*p*=0.37) or ≥1.1ng/mL (*p*=0.62) (Figure 1). There was also no correlation between TSH and AFC (*p*=0.56).

## DISCUSSION

Ovarian reserve can be assessed by evaluating serum FSH, serum AMH and AFC, the latter two are more reliable and, therefore, more frequently used (Cedars, 2022). AMH measurement is currently considered the most accurate indicator for low ovarian reserve diagnosis, with an established cutoff <1.1 ng/mL, according to the *Brazilian Society* of *Human Reproduction* consensus (Miklos *et al.*, 2019), despite the possibility of inter-cycle variations (Melado *et al.*, 2018); while AFC is considered the most subjective tool, as it is dependent examiner. It is well known that thyroid dysfunction has a negative impact on fertility and pregnancy (Tańska *et al.*, 2022), but the association between thyroid autoimmunity and ovarian reserve has been little studied and data from available studies are still controversial (Hasegawa *et al.*, 2021). A study by Wei *et al.* (2023) found that the AMH levels of TPOAb-positive patients were significantly lower than those found in patients with negative TPOAb, indicating that this last group had a higher ovarian reserve. On the other hand, in agreement with our findings, Polyzos *et al.* (2015) evaluated 4.894 women and did not find an association between thyroid autoimmunity/hypothyroidism and low ovarian reserve assessed by serum AMH. Moreover, Wei *et al.* (2023) also divided patients into 2 groups of positive TPOAb levels (high and low titers) and found no significant difference in the clinical pregnancy rate between them, but the live birth rate of the high-titer TPOAb group was significantly lower than that of the low-titer group.

A recent metanalysis by Hasegawa *et al.* (2021) demonstrated that AMH levels tended to decline with thyroid autoimmunity in euthyroid adults; however, AMH levels were substantially higher in euthyroid adolescents with antithyroid antibodies. Additionally, Morales-Martínez *et al.* (2021) found that women with autoimmune hypothyroidism (in euthyroidism, under levothyroxine replacement) had no difference in either AMH levels or AFC, when compared to healthy women, concluding that there was no significant correlation between thyroid autoimmunity and impaired ovarian reserve.

It is well established that female sex is a strong determinant of TPOAb positivity (Keyhanian *et al.*, 2019) and that this antibody has been associated with other autoimmune diseases (Nakamura *et al.*, 2008). Therefore, detectability of TPOAb has been studied to evaluate its association with nonthyroidal diseases (Khan *et al.*, 2022; Meneghini *et al.*, 2024). TPOAb detectability, rather than positivity, was associated with an increased risk of all-cause, cardiovascular, and cancer-related causes, especially in men (Khan *et al.*, 2022). Moreover, there are reports of antithyroid autoantibody positivity related to postpartum depression in women; however, the results are heterogeneous and inconclusive (Sileo *et al.*, 2023). In our cohort, 14% of the patients had positive TPOAb, similar to the ELSA-Brasil multicentric study, which demonstrated 12% positivity (Meneghini *et al.*, 2024). In this study, sociodemographic and lifestyle-related factors were determinants of TPOAb detectability, positivity, and persistent positivity, as well as the impact of TPOAb on mortality risk. Therefore, we stratified TPOAb and TgAb in numerical categories of detectability and found no association between AMH and detectable categories of TPOAb or TgAb. Both antithyroid antibodies levels were not statistically different between women with normal and low ovarian reserve assessed by AMH levels or by AFC. Even after adjusting for chronological age and endometriosis, the most prevalent cause of consultation in the assisted reproductive clinic observed in our sample, we did not find any association between AMH and detectable categories of antithyroid antibodies. We did not determine an association between antithyroid detectability and AFC, as it is a less precise and examiner-dependent test.

As the studied women visited the assisted reproduction clinic for different reasons, including oocyte cryopreservation, we stratified them according to ovarian reserve evaluated by AMH levels and AFC. Antithyroid antibodies also did not differ between the low and normal ovarian reserve groups evaluated using AMH and AFC.

Interestingly, our study provides data regarding serum TgAb levels, which are rarely available in similar studies. Unuane *et al.* (2013) previously demonstrated that 5% of subfertile women had isolated positive TgAb, while 4% showed isolated positive TPOAb. The authors also reported a significantly higher median serum TSH levels in women with positive thyroid antibodies than those without thyroid autoimmunity.

In the current study, there was no correlation between TSH and AMH levels even after adjusting AMH levels according to the Brazilian criteria of low ovarian reserve. Similarly, AFC did not demonstrate a correlation with TSH levels in women with follicle count ≥7 or in those with low ovarian reserve.

Even though it was not the purpose of our study, we evaluated the correlation between BMI and serum AMH, since an inverse association between them has already been reported (Moslehi *et al.*, 2018; di Clemente *et al.*, 2021). Nevertheless, we did not find a statistically significant correlation between BMI and AMH levels.

Our data endorses the medical literature regarding the protective effect of younger age in ovarian reserve; however, it does not consolidate evidence on the diminished ovarian reserve associated with endometriosis (Tan *et al.*, 2023a) as it is well known that endometriosis disrupts normal follicular activation and maturation, contributes to ovarian dysfunction, and reduces the number of ovarian follicles (Tan *et al.*, 2023b). Women with endometriosis, particularly those of advanced age, are at a heightened risk of reduced ovarian reserve compared with their younger, healthy counterparts (Tan *et al.*, 2023a; Wang *et al.*, 2023). These discrepant results may be related to many factors, including the median age of our cohort, the methodology used to assess ovarian reserve and endometriosis, and the severity and location of the endometriosis foci.

Finally, it is important to highlight that although our results suggest that antithyroid antibody detectability is not significantly associated with ovarian reserve, the evaluation of thyroid autoimmunity in early pregnant women seems relevant. Andersen *et al.* (2022) recently established lower pregnancy-specific cut-offs for TPOAb and especially for TgAb in 10,905 early pregnant women. Antibody-positive women (TPOAb and/or TgAb) had higher median TSH levels and were more likely to have hypothyroidism in early pregnancy and be diagnosed with hypothyroidism during follow-up. Therefore, this study supports the indication of thyroid autoimmunity assessment, and that the positivity cut-off defined by the assay manufacturer should be evaluated with caution in pregnant women (Andersen *et al.*, 2022).

Our study had some limitations. As this was an observational study based on medical records, the presence of biases related to data collection cannot be excluded. Furthermore, it is known that serum antibody concentrations may vary; however, both TPOAb and TgAb were assessed in a single blood sample. Antithyroid antibodies were measured only in one visit; 43.3% of patients had low detectable TPOAb, and 66.9%, low detectable TgAb. We cannot confirm if a second measurement of these antibodies would be in the same category or have an undetectable or highly detectable result. The same justification can be made for negative values.

In conclusion, we found no association between the detectability or positivity of TPOAb and/or TgAb and ovarian reserve, assessed by serum AMH levels, in women who visited an assisted reproductive clinic. Moreover, we did not find a correlation between AMH and TSH or between AFC and TSH. These results suggest that neither the detectability nor the positivity of antithyroid antibodies influence ovarian reserve, assessed by AMH.
